# Subjective Oral Health Perception and Oral Health Behavior in of Korean Adolescents

**Published:** 2020-01

**Authors:** Yong-Keum CHOI, Jin-Sun CHOI

**Affiliations:** 1 Department of Dental Hygiene, College of Health Science and Genome-Based BioIT Convergence Institute, Sun Moon University, Asan, South Korea; 2 Department of Preventive and Public Health Dentistry, College of Dentistry and Research Institute of Oral Science, Gangneung-Wonju National University, Gangneung, South Korea

## Dear Editor-in-Chief

The subjective oral health perception refers to the oral health condition a person perceives himself/herself. A positive motivation may be given for personal oral health management. There is a relationship between subjective oral health perception, oral health conditions and oral health behaviors (1–3). And the subjective health level is a useful way to easily and simply assess adolescents’ health (4). For Korean adolescents who spend most of their time in school and are unable to use dental institutions, therefore, the subjective health perception index with which Korean adolescents can have self-oral health conditions and detect their behaviors must be used.

This study aimed to examine the oral health behavior according to the subjective oral health perception in Korean adolescents, and investigate the relationship between the subjective oral health perception and variables to find out whether the subjective oral health perception index may be appropriate to detect oral health and behaviors of adolescents. In this study, 12th (2016) Korean Youth Health Risk Behavior Online Survey was used as the original source. The subjective oral health perception was selected as a dependent variable to use independent variables for demographic factors and oral health-related behavior factors in the analysis. To identify the statistical significance between the subjective oral health perception and relevant factors, complex sample Chi-square test was employed. Moreover, the complex sample multi-variate logistic regression model was used to verify the relationship between subjective oral health perception and oral health behavior. Statistical analysis were done through STATA 13.0 (Copyright Stata Corp LP, USA)

When looking at the subjective oral health perception of Korean adolescents, ‘Healthy’ was 42.3%, ‘Average’ was 41.0%, and ‘Not healthy’ was 16.7%. Depending on the subjective oral health perception, the factors of oral health behavior was statistically significant (*P*<0.001) (Table 1). In addition, variables related to the subjective oral health perception were found to be ‘Toothbrushing in a day’, ‘Toothbrushing after lunch at school’, ‘The number of oral care products’, ‘Experience of sealant’, ‘Oral symptoms’, ‘Fruit intake’, ‘Vegetable intake’, ‘Sweet flavor drink intake’, and ‘Carbonated drink intake’ (*P*<0.001) (Table 2).

**Table 1:** The distribution of subjects who have subjective oral health perception

***Classification***		***N***	***Subjective oral health perception***						**P-*value***
			***Healthy***		***Normal***		***Poor***		
			***N***	***Wt%^†^***	***N***	***Wt%^†^***	***N***	***Wt%^†^***	
Total		65,528	27,923	42.3	26,762	41.0	10,843	16.6	
Daily tooth brushing	No	690	135	18.5	266	39.2	289	42.2	<0.001
	=1~2	32,930	12,326	37.3	14,309	43.5	6,295	19.1	
	≥3	31,908	15,462	48.0	12,187	38.3	4,259	13.5	
Tooth brushing after lunch at school	Always	16,204	7,857	48.2	5,957	36.9	2,390	14.8	<0.001
	Sometimes	24,135	10,180	41.8	10,073	41.7	3,882	16.3	
	No	25,189	9,886	39.1	10,732	42.8	4,571	18.0	
Using oral hygiene products	No	37,197	15,028	40.1	15,509	41.9	6,660	17.9	<0.001
	=1~2	26,882	12,119	44.7	10,767	40.1	3,996	15.1	
	≥3	1,449	776	53.2	486	33.2	187	13.4	
Experience of sealant	Yes	47,517	21,030	43.9	19,262	40.8	7,225	15.2	<0.001
	No	18,011	6,893	38.1	7,500	41.6	3,618	20.2	
Experience of scaling	Yes	49,738	21,300	42.4	20,437	41.3	8,001	16.2	<0.001
	No	15,790	6,623	41.8	6,325	40.2	2,842	17.9	
Oral symptoms	Yes	26,727	15,055	56.8	9,839	37.1	1,833	6.9	<0.001
	No	38,801	12,868	33.0	16,823	43.6	9,010	23.2	
Fruit intake	No	5,729	1,913	32.6	2,495	44.2	1,913	23.1	<0.001
	≥1/week	44,804	18,392	40.7	18,879	42.2	18,392	16.9	
	≥1/day	14,995	7,618	50.4	5,388	36.2	7,618	13.3	
Vegetable intake	No	2,452	761	31.2	13,614	43.3	637	25.3	<0.001
	≥1/week	35,244	1,054	38.4	15,407	43.7	6,223	17.8	
	≥1/day	27,832	637	48.2	10,301	37.3	3,983	14.3	
Milk intake	No	10,308	3,821	37.1	4,394	42.4	3,821	20.3	<0.001
	≥1/week	37,076	15,351	41.1	15,555	42.2	15,351	16.6	
	≥1/day	18,144	8,751	47.9	6,813	37.6	8,751	14.4	
Carbonated drink intake	No	15,895	7,236	45.5	6,196	39.0	2,463	15.4	<0.001
	≥1/week	47,037	19,717	41.4	38.98	41.7	7,796	16.7	
	≥1/day	2,956	970	37.8	19,524	39.5	584	22.6	

The data were analyzed by Chi-square test,

† Weighted value

**Table 2:** Odds ratios of subjective oral health perception on health among groups categorized by factors. OR (95% Cl)

***Classification***		***Model 1***	***Model 2***
Fruit intake	Once a day and more	Ref. 1.000	Ref. 1.000
	Once a week and more	0.692(0.806–0.865)^‡^	0.772(0.741–0.804)^‡^
	No	0.482(0.453–0.514)^‡^	0.590(0.545–0.638)^‡^
Vegetables intake	Once a day and more	Ref. 1.000	Ref. 1.000
	Once a week and more	0.680(0.658–0.703)^‡^	0.716(0.688–0.745)^‡^
	No	0.500(0.457–0.547)^‡^	0.536(0.480–0.599)^‡^
Milk intake	Once a day and more	Ref. 1.000	Ref. 1.000
	Once a week and more	0.881(0.849–0.913)^‡^	0.826(0.792–0.862)^‡^
	No	0.679(0.664–0.733)^‡^	0.753(0.711–0.798)^‡^
Carbonated beverage intake	No	Ref. 1.000	Ref. 1.000
	Once a week and more	0.829(0.797–0.861)^‡^	0.831(0.795–0.869)^‡^
	Once a day and more	0.692(0.634–0.755)^‡^	0.678(0.608–0.757)^‡^
After lunch Toothbrushing	Always	Ref. 1.000	Ref. 1.000
	Sometimes	0.769(0.739–0.801)^‡^	0.685(0.654–0.719)^‡^
	No	0.695(0.665–0.726)^‡^	0.578(0.550–0.607)^‡^
Daily tooth brushing	≥3	Ref. 1.000	Ref. 1.000
	=1~2	0.603(0.583–0.624)^‡^	0.643(0.620–0.668)^‡^
	No	0.222(0.185–0.267)^‡^	0.212(0.164–0.275)^‡^
Using oral hygiene assistant products	≥3	Ref. 1.000	Ref. 1.000
	=1~2	0.742(0.667–0.826)^‡^	0.788(0.695–0.892)^‡^
	No	0.628(0.564–0.698)^‡^	0.721(0.636–0.816)^‡^

The data were analyzed by Complex Samples Logistic Regression

* *P*<0.05,

† *P*<0.01,

‡ *P*<0.001

Model 1: Adjusting for Gender and Grade

Model 2: Adjusting for Gender, Grade, Fathergistic Regressionong groups categorized by factorsunhealthy to have a positive percep

Since there is a significant relationship between the subjective oral health perception and behaviors related to oral health. For adolescents, subjective health levels perceived as a constant self-concept rather than an immediate assessment of their health. This is very important because it leads to health-related identity in adulthood (5). Therefore, the subjective oral health perception index is an appropriate index to find out the oral health behaviors of adolescents. An oral health improvement program is considered necessary for adolescents who think they are unhealthy to have a positive perception of their oral health.
